# The Influence of Enzymatic Hydrolysis on Bee Pollen Antioxidant and Antibacterial Activities

**DOI:** 10.3390/foods12193582

**Published:** 2023-09-26

**Authors:** Vaida Damulienė, Vilma Kaškonienė, Paulius Kaškonas, Audrius Maruška

**Affiliations:** 1 Instrumental Analysis Open Access Centre, Vytautas Magnus University, LT-44404 Kaunas, Lithuania; vaida.damuliene@vdu.lt (V.D.); audrius.maruska@vdu.lt (A.M.); 2 Institute of Metrology, Kaunas University of Technology, LT-51368 Kaunas, Lithuania; paulius.kaskonas@ktu.lt

**Keywords:** pollen, hydrolysis, enzymes, antioxidant activity, antibacterial activity, chemometric analysis

## Abstract

Bee pollen is one of the most valuable apitherapeutic products with high nutritional value. To obtain a higher diversity of compounds, higher bioactivity, and improve the release of nutrients from bee pollen, additional processing of the raw material may be applied: fermentation using microorganisms or hydrolysis using selective enzymes. This research aimed to determine the impact of enzymatic hydrolysis on the antioxidant and antibacterial activities of bee pollen. Bee pollen samples from Sweden, Spain, Netherlands, Italy, Poland, Denmark, Slovakia, Malta, and Lithuania were hydrolyzed using pure enzymes, including lipase, cellulase, protease, and amyloglucosidase, as well as enzyme mixtures such as *Viscozyme^®^ L* and *Clara-diastase*. Total phenolic content, total flavonoid content, and antioxidant activity were analyzed spectrophotometrically. Antibacterial activity against *Staphylococcus aureus*, *Listeria monocytogenes*, *Staphylococcus enteritidis*, and *Salmonella typhimurium* was evaluated using the agar well diffusion assay. Obtained results revealed a positive effect of enzymatic hydrolysis on biologically active compound content and activity: total phenolic content increased by 1.1 to 2.5 times, total flavonoid content by 1.1 to 3.0 times, radical scavenging activity by 1.1–3.5 times, and antibacterial activity by 1.1 to 3.3 times. K-means clustering analysis grouped samples into 5–9 clusters and was dependent on the measured characteristic used as an input—total phenolic compounds content, total flavonoid content, antioxidant activity, and antibacterial activity against four different bacteria. Chemometrics showed, that the enzyme used for the hydrolysis had a higher impact on clustering results than the geographical origin of the samples.

## 1. Introduction

Biologically active compounds, such as fatty acids, phenolic acids, and carotenoids, are well-known agents for promoting health. In recent years, there has been a growing interest in obtaining functional ingredients from natural sources, especially bee pollen. Nowadays, bee pollen is considered an alternative food supplement with anticarcinogenic, immune-stimulating, antioxidant, anti-inflammatory, and antimicrobial functions due to its unique phytochemical composition [1,2].

Bee pollen is protected by a strong inner layer from cellulose and pectin called intine and an outer layer from sporopollenin—exine [3]. This double-layer structure depends on the botanical and geographical origin of bee pollen and affects the product resistance against microbial contamination, temperature, UV, or pH changes [4]. Moreover, the bee pollen complex membrane negatively interferes with specific enzymes involved in digestion in the human gastrointestinal tract reducing bioaccessibility [3,5].

A high interest in improving existing methods to increase the absorption of bee pollen substance in the simplest possible way has emerged in the last few years. The earliest methods included chemical treatment with monoethanolamine, mechanical treatment with the action of shear forces generating heat [6,7], physical treatment with supercritical fluids, and ultrasound [7,8]. However, these approaches were unacceptable due to nutritional loss or unusability in the food industry. Recently, several studies have shown promising results achieved by using biotechnological processes to improve the release of bee pollen nutrients to the human body. Research using fermentation with lactic acid bacteria or enzymatic hydrolysis has revealed that the treatment successfully destroys bee pollen layers, and increases the amount of biologically active compounds together with an increment in antimicrobial or antioxidant activities [6,9,10,11,12,13].

The aim of this research is to determine the impact of enzymatic hydrolysis on the antioxidant and antibacterial activities of bee pollen from various European regions. Acquired data will help properly determine the prospective method allowing us to obtain a higher diversity of compounds, higher bioactivity, and improve the release of bee pollen biologically active compounds to human organisms.

The success of enzymatic hydrolysis depends on a combination of several factors, including enzyme selection and concentration, substrate characteristics and reaction conditions. Each system may require specific considerations and optimizations to achieve the desired outcomes. To the best of our knowledge, this is the first study to explore the optimization of enzymatic hydrolysis duration, enzyme concentration, and substrate pH to enhance the antioxidant and antibacterial activity of bee pollen. Furthermore, there is limited literature available on the antibacterial activity of enzymatically treated bee pollen.

## 2. Materials and Methods

### 2.1. Bee Pollen Samples and Extract Preparation

**Figure 1:**
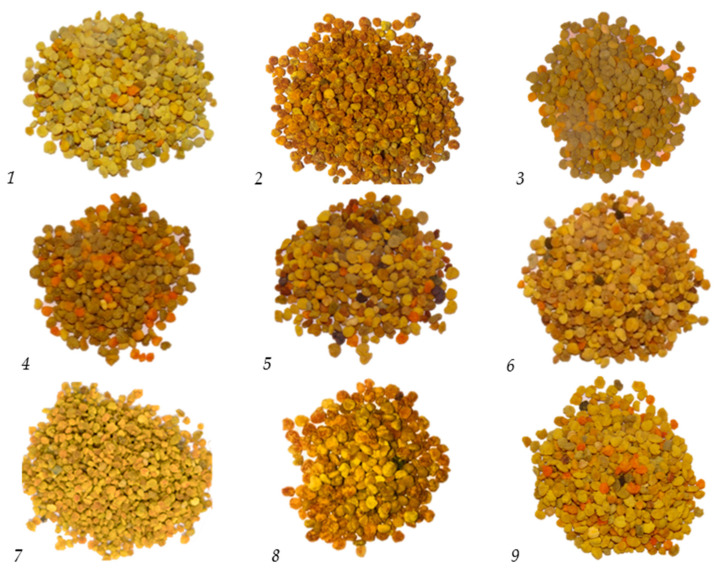
Bee pollen samples from different countries of Europe (1—Denmark, 2—Sweden, 3—The Netherlands, 4—Republic of Malta, 5—Spain, 6—Italy, 7—Lithuania, 8—Poland, 9—Slovakia).

Bee pollen samples from nine European regions were used in this study (see Table 1, Figure 1). Pollen was collected during the flowering season from May to August in 2018. Dried pollen samples were stored at +5 °C for a maximum of four weeks and homogenized with a pestle and porcelain mortar before extract preparation. Methanolic pollen extracts were prepared for spectrophotometric and antibacterial analysis according to Kaškonienė et al. [9], and aqueous extracts were prepared for oxidation-reduction potential evaluation according to Adaškevičiūtė et al. [14].

### 2.2. Chemicals and Reagents

Hexamethylenetetramine (≥99%), aluminum chloride (≥98%), agar (Agar-Agar), and LB broth were obtained from Carl Roth Gmbh & Co Kg (Karlsruhe, Germany). The 2,2-diphenyl-1-picrylhydrazyl (DPPH) (99%), methanol (≥99.9%), rutin (95%), *Viscozyme^®^ L*, cellulase from *Aspergillus niger*, *Clara-diastase*, lipase from *Aspergillus oryzae*, protease from *Bacillus* species, amyloglucosidase from *Aspergillus niger*, Tween 20 broth, trichloroacetic acid (≥99%), starch (soluble) and 3,5-dinitrosalicylic acid (DNS) were obtained from Sigma-Aldrich Corporation (Taufkirchen, Germany). Folin–Ciocalteu reagent, casein and acetonitrile (≥99.8%) were supplied by Merck (Darmstadt, Germany). Sodium carbonate, calcium carbonate, and acetic acid (99.9%) were bought from Reachem S.r.o. (Bratislava, Slovakia). Sodium acetate (trihydrate) (99%) was obtained from ChemPur (Piekary Śląskie, Poland). Cellulose and ceftazidime pentahydrate (98%) were obtained from Acros Organics (Geel, Belgium). Bidistilled water was prepared by means of the distillation apparatus Thermo Scientific (Fremont, CA, USA).

### 2.3. Determination of Enzymes Optimal Conditions

Enzymes are active only at optimal pH and temperature. However, manufacturers precisely specify the optimal temperature but the pH range is quite wide (Table 2). There were prepared sodium acetate and sodium phosphate buffers for analysis at different pH intervals. A 0.1 M sodium acetate buffer, regulated with 0.1 M acetic acid, was prepared for the pH interval of 3.6–5.6. This buffer was used to determine the optimal pH for amyloglucosidase, cellulase, and *Viscozyme^®^ L* enzymes. For the pH interval of 5.8–7.8, a 0.1 M sodium phosphate buffer, regulated with 0.1 M disodium phosphate, was used. This buffer was employed to evaluate the optimal pH for *Clara-diastase*, lipase, and protease. Samples were prepared according to procedures described in Table 3. Accurate pH values for optimal conditions for enzymes were determined according to the highest substrate concentration measured using a Hipo MPP-96 spectrophotometer (Biosan Laboratories, Latvia) at a specific wavelength indicated in Table 3.

### 2.4. Enzymatic Hydrolysis of Bee Pollen

For enzymatic hydrolysis six commercial enzymes were utilized: *Clara-diastase*, *Viscozyme^®^ L*, amyloglucosidase, protease, lipase, and cellulase. The bioprocess was conducted in 10 mL glass vials, following the procedure outlined by Zuluaga-Dominguez et al. [12], with slight modifications. To begin, 1 g of each bee pollen sample was moistened with 0.5 mL of sterile bidistilled water and heated for 15 min at a temperature of +121 °C. Subsequently, 0.05 U of each enzyme was dissolved in an optimal pH buffer (see Section 3.1). The moistened bee pollen was mechanically mixed with the determined optimal enzyme amount and the mixture was incubated for the determined optimal duration for enzymatic hydrolysis (see Section 3.2). The bioprocess was terminated by boiling the vials for 2 min. After enzymatic hydrolysis, the solid state of the sample was extracted using 80% methanol. Control bee pollen samples, which served as natural pollen samples before enzymatic hydrolysis, were prepared using the same procedure replacing the enzyme with a buffer of corresponding pH.

An optimal enzymatic hydrolysis duration was assessed by conducting the process for 1, 2, 3, 4, and 5 h. An optimal amount of enzyme was determined by using 50, 100, 150, 200, and 300 µL of each enzyme. A data set was created measuring the following four parameters of bee pollen samples from Lithuania ten times: total phenolic content (TPC), total flavonoid content (TFC), radical scavenging activity (RSA), and oxidation-reduction potential. Differential calculations of the obtained data were performed as follows: data points corresponding to each measured parameter were approximated and interpolated. Subsequently, derivatives were calculated from the resulting curves. Identifying the maximum values of the derivative curves allowed us to find the optimal enzyme quantity and the optimal duration of enzymatic hydrolysis.

### 2.5. Oxidation-Reduction Potential

The oxidation-reduction potential was determined following the method described by Alwazeer and Sally [19]. Natural and enzyme-hydrolyzed aqueous extracts of bee pollen were analyzed with a multimeter using a combined redox electrode Benchtop Meter DHS (XS Instruments, Reicholzheim, Germany). The pH of the samples was measured with a pH-meter UltraBasic Benchtop UB-10 (Denver Instrument Company, Denver, CO, USA) using a glass electrode. Oxidation-reduction potential (ORP) values in the samples were calculated using Equation (1):

ORP = Eh − 59·(7 − pH),(1)

where Eh is the measured electrode potential value (mV), pH is the sample pH value.

### 2.6. Spectrophotometric Evaluation

Spectrophotometric evaluation of natural and enzymatically hydrolyzed bee pollen was carried out. Briefly, the amount of total phenolic compounds was determined by the colorimetric method of Folin–Ciocalteu. Total flavonoid content was measured using AlCl_3_ colorimetric stock solution. Radical scavenging activity was determined by employing a 2,2-diphenyl-1-picrylhydrazyl (DPPH) free radical colorimetric reaction. A more detailed description of the methods used for the determination of total phenolic compounds, total flavonoid content, and radical scavenging activity can be found in Adaškevičiūtė et al. [14]. Successive analyses were conducted using a Hipo MPP-96 spectrophotometer (Biosan Laboratories, Latvia) expressing the results as mg of rutin equivalent (RUE) per 1 g of raw material.

### 2.7. Antibacterial Evaluation

The antibacterial activity of natural and enzymatically hydrolyzed bee pollen methanolic extracts was determined using the agar well diffusion method against Gram-positive *Staphylococcus aureus*, *Listeria monocytogenes*, and Gram-negative *Salmonella enteritidis*, *Salmonella typhimurium* bacteria cultures. The detailed methodology was described in Adaškevičiūtė et al. [20]. Briefly, 100 µL of an overnight-grown bacterial suspension was spread evenly onto Petri dishes with sterile LB-agar. In each dish, five wells were cut, and the bottom was covered with a drop of agar. 100 µL of each natural and enzymatically hydrolyzed bee pollen extract was added to three wells, while the remaining two were filled with 80% methanol and 0.9% NaCl. The plates were incubated for 24 h at a temperature of +37 °C and the clear zones around the wells were measured. The results were expressed as µg ceftazidime equivalent (CEF) per 1 mL of bee pollen extract.

### 2.8. Statistical Analysis

All spectrophotometric measurements were performed 10 times. Data were organized using MS Excel 15.11.2 (2015, Microsoft, Redmond, Washington, DC, USA) software and the results were analyzed using linear regression modeling. Successive chemometric analysis was performed using MATLAB v9.1.0 (R2016b, MathWorks, Natick, MA, USA) software.

Before data mining standardization of the results was performed by subtracting the means of the corresponding variables and dividing by their standard deviations. Statistical analysis involved the use of analysis of variance (ANOVA), k-means clustering analysis (k-means), and analysis of correlation. Clustering analysis was employed to assess similarities among all bee pollen samples and group them into clusters based on the measured characteristics. Because the k-means technique requires the presentation of the number of clusters as input, the Davies–Bouldin index, Calinski–Harabasz criterion, Silhouette, and distortion function were evaluated as decision criteria [21]. Hypotheses regarding the equalities of measured parameters means among tested bee-pollen samples from various Europe regions were tested using ANOVA, with a selected level of significance *p* ≤ 0.05. The relationship between measurands was evaluated using Pearson’s linear correlation coefficient at the same significance level *p* ≤ 0.05.

## 3. Results and Discussion

Nine samples of bee pollen underwent hydrolysis using lipase, protease, cellulase, *Clara-diastase*, *Viscozyme^®^ L*, and amyloglucosidase. The impact of enzymatic hydrolysis was assessed by comparing total phenolic compounds, flavonoid content, radical scavenging, and antibacterial activity before and after hydrolysis. To the best of our knowledge, this is the first study to optimize the duration and amount of enzymes used in the enzymatic hydrolysis of bee pollen.

### 3.1. Determination of Optimal pH for Enzymes Activity

**Figure 2:**
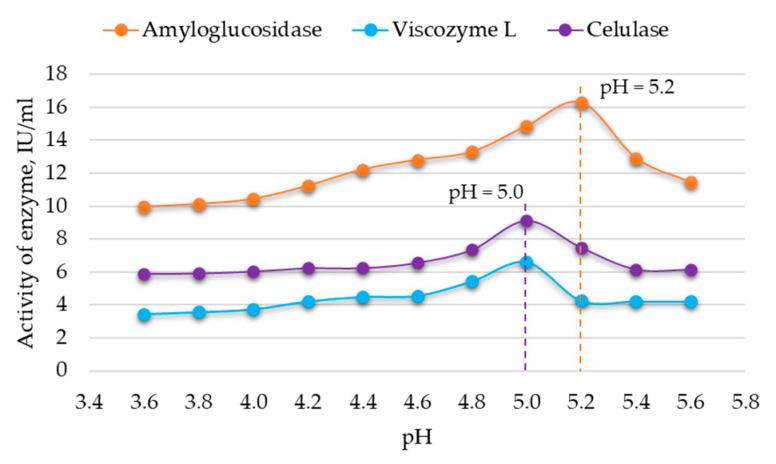
Changes in activity of enzymes at pH 3.6–5.6 (RSD ≤ 0.6%, n = 10).

**Figure 3:**
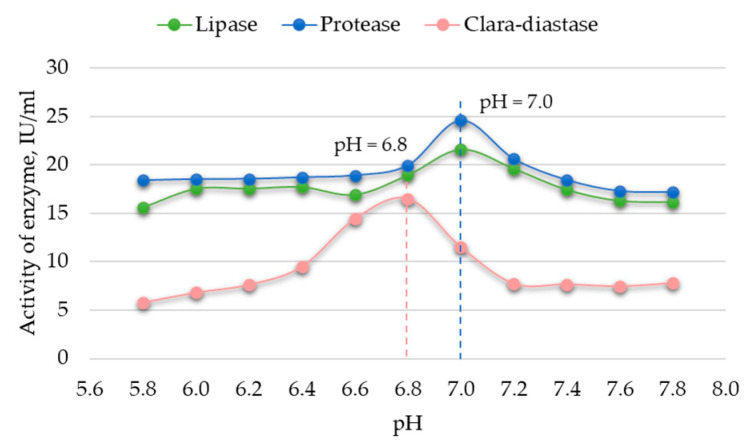
Changes in activity of enzymes at pH 5.8–7.8 (RSD ≤ 0.7%, n = 10).

The relationship between enzymatic activity and substrate pH was determined following the manufacturer’s recommendations (see Table 2). Enzymatic activity was controlled within the pH ranges of 3.6–5.6 and 5.8–7.8 using sodium acetate and sodium phosphate buffers. The results are presented in Figure 2 and Figure 3.

The data indicate that the optimal pH for achieving the highest activity of *Viscozyme^®^ L* and cellulase is 5.0, while for amyloglucosidase, the optimal pH is 5.2 (Figure 2). An optimal pH value of 6.8 is identified for achieving the highest activity of *Clara-diastase*, and a pH of 7.0 is optimal for the highest activity of lipase and protease (Figure 3). Uchida and Santos [22] also determined that the best activity of cellulase is achieved at pH 5. Furthermore, *Viscozyme^®^ L* is identified as a cellulolytic enzyme mixture, with its maximum activity observed at the same pH. A similar optimal pH for amyloglucosidase was reported by Malik et al. [23]. Additionally, studies by Xiang et al. [24] for lipase activity and Otroshi et al. [25] for protease activity indicated a maximum activity at pH 7. The determined optimal pH conditions for the enzymes were employed for further experiments with bee pollen.

### 3.2. Optimization of Conditions for Bee Pollen Enzymatic Hydrolysis

The optimization of enzymatic hydrolysis duration was evaluated using a randomly chosen Lithuanian pollen sample. The chosen sample was enzymatically hydrolyzed with six commercial enzymes: *Clara-diastase*, *Viscozyme^®^ L*, amyloglucosidase, protease, lipase, and cellulase. The optimization of enzymatic hydrolysis involved the comparison of TPC, TFC, RSA, and oxidation-reduction potential after 1, 2, 3, 4 and 5 h of the hydrolysis process, utilizing 50, 100, 150, 200 and 300 µL of each enzyme.

The study revealed that enzymatic hydrolysis had a positive effect on increasing the content of biologically active compounds. However, the extent of the increase depended on both duration and the ratio of bee pollen to enzyme. The amounts of measured compounds in bee pollen samples significantly increased after exposure to different enzymes: TPC increased by 1.3–2.3 times (*p* ≤ 0.05), TFC increased by 1.2–2.2 times (*p* ≤ 0.05), and RSA increased by 1.3–1.8 times (*p* ≤ 0.05). Oxidation-reduction potential indicated a significant variation between samples, ranging from 133.68 to 195.00 mV (*p* ≤ 0.05). The impact of the bee pollen to enzyme ratio was tested by altering the volume of the enzyme solution. A significant increase in the total phenolic compounds content by 1.3–2.3 times was observed in the tested samples (*p* ≤ 0.05). The increase of evaluated TFC after enzymatic hydrolysis of bee pollen was statistically significant and ranged from 1.3 to 1.8 times at *p* ≤ 0.05. Based on the obtained results, it was determined that RSA increased from 9.23 ± 0.03 to 16.01 ± 0.03 mg RUE/g and correlated with total phenolic content (0.816 at *p* ≤ 0.05). Oxidation-reduction potentials varied from 133.68 to 203.00 mV in bee pollen samples after the addition of different volumes of enzymes.

**Figure 4:**
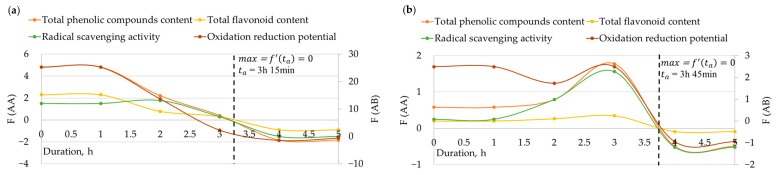
Determination of the optimal duration of enzymatic hydrolysis using interpolation (F(AA) is the variation of TPC, TFC, and RSA in mg RUE/g per hour, F(AB) is the variation of oxidation-reduction potential mV per hour based on: (**a**) cellulase, *Viscozyme^®^ L*, and *Clara-diastase* results; (**b**) protease, lipase, and amyloglucosidase results).

**Figure 5:**
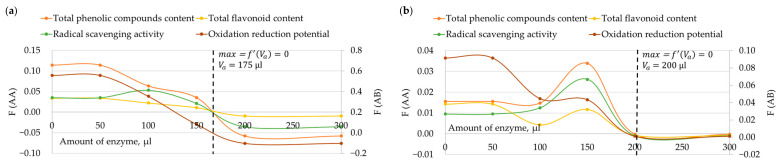
Determination of the optimal amount of enzyme for enzymatic hydrolysis using interpolation (F(AA) is the variation of TPC, TFC, and RSA in mg RUE/g per hour, F(AB) is the variation of oxidation-reduction potential mV per hour based on: (**a**) cellulase, *Viscozyme^®^ L*, and *Clara-diastase* results; (**b**) protease, lipase, and amyloglucosidase results).

The determination of the optimal amount of enzyme and the optimal duration of enzymatic hydrolysis was grounded on the alterations in TPC, TFC, RSA, and oxidation-reduction potential as previously described. Approximated functional dependencies for each parameter were found and derivatives were derived. Subsequently, parameter derivative curves were plotted after interpolation. The presentation of the optimal duration of enzymatic hydrolysis is depicted in Figure 4, while the optimal amount of enzyme is illustrated in Figure 5.

The determination of the optimal amount of enzyme and the optimal duration of enzymatic hydrolysis for each specific tested enzyme was based on derivative curves of each parameter function. The maximum point of each parameter corresponded to the point where the magnitude of the derivative curve changed from positive to negative upon crossing the x-coordinate axis.

Based on the previously described derivatives of TPC, TFC, RSA, and oxidation-reduction potential, it was determined that the optimal duration of enzymatic hydrolysis using cellulase, *Viscozyme^®^ L* and *Clara-diastase* is 3 h 15 min, whereas for protease, lipase and amyloglucosidase it is 3 h 45 min (Figure 3). The optimal amount of cellulase, *Viscozyme^®^ L*, and *Clara-diastase* for enzymatic hydrolysis was found to be 175 µL, while for protease, lipase, and amyloglucosidase, it was 200 µL (Figure 4). A shorter duration of enzymatic hydrolysis and a lower amount of cellulase, *Viscozyme^®^ L*, and *Clara-diastase* can be considered for bee pollen hydrolyzation. *Viscozyme^®^ L* and *Clara-diastase* enzyme mixtures include cellulase. Bee pollen contains cellulose in the inner layer of the cell wall, which can be easily broken down by cellulase, potentially leading to the release of biologically active compounds [8].

### 3.3. Variation of Total Phenolic Compounds, Flavonoid Content, and Radical Scavenging Activity in Natural and Enzymatically Hydrolyzed Bee Pollen

The extracts were prepared from nine bee pollen samples originating different regions of Europe, including Sweden, Spain, Netherlands, Italy, Poland, Denmark, Slovakia, Malta, and Lithuania. The hydrolysis process was applied using lipase, cellulase, protease, amyloglucosidase, *Viscozyme^®^ L*, and *Clara-diastase* enzymes. The impact of the enzymatic hydrolysis was assessed by comparing changes in TPC, TFC, and RSA before and after the enzymatic hydrolysis process, and statistically significant (*p* ≤ 0.05) variations are presented in Table 4.

Enzymatic hydrolysis under optimized conditions resulted in an increase in TPC by 1.1 to 2.5 times at a significance level of *p* ≤ 0.05. Prior to fermentation, all bee pollen samples exhibited TPC levels ranging from 8.07 to 12.67 mg RUE/g. After hydrolysis with protease, TPC changed from 23.6 to 94.4%; lipase from 6.8 to 41.9%; cellulase from 55.8 to 150.0%; *Clara-diastase* from 36.0 to 113.9%; *Viscozyme^®^ L* from 21.3 to 135.0% and amyloglucosidase from 1.5 to 34.8%. According to the results, the increase in TPC was dependent on the geographical origin of bee pollen. Given that geographic location is correlated with climate, it can reasonably be assumed that bee pollen accumulates a higher quantity of biologically active substances in cooler regions, leading to a greater release during enzymatic hydrolysis [26]. The findings revealed that bee pollen from the northern part of Europe exhibited a higher TPC compared to pollen from the south, with a statistically significant difference ranging from 1.1 to 2.0 times (*p* ≤ 0.05).

Furthermore, the amount of TPC obtained after enzymatic hydrolysis of bee pollen with cellulase, *Viscozyme^®^ L* and *Clara-diastase* was 19.8 to 55.5% higher compared to hydrolysis with protease, amyloglucosidase, and lipase. The highest TPC was determined after hydrolysis with cellulase (ranging from 12.57 ± 0.02 to 31.67 ± 0.05 mg RUE/g), while the lowest was observed with amyloglucosidase (ranging from 8.84 ± 0.09 to 16.17 ± 0.05 mg RUE/g) (Table 4). A higher quantity of TPC was determined after enzymatic hydrolysis with *Viscozyme^®^ L* and *Clara-diastase* due to the presence of cellulase in their composition. The greater increase in biologically active substances could be attributed to the cellulase and the mixture’s ability to break down the pollen cell wall, which consists of an external layer made of sporopollenin and an internal layer composed of pectin and cellulose [1,27]. TFC also significantly (*p* ≤ 0.05) increased by 1.1 to 3.0 times after treatments with all tested enzymes (see Table 4). The results revealed that bee pollen enzymatically hydrolyzed with cellulase, *Viscozyme^®^ L* and *Clara-diastase* exhibited 1.5 to 2.5 times higher flavonoid content compared to hydrolysis with lipase, protease, and amyloglucosidase. Furthermore, bee pollen hydrolyzed with cellulase released the highest flavonoid content (ranging from 6.46 ± 0.06 to 15.83 ± 0.05 mg RUE/g), while the lowest results were observed with amyloglucosidase (ranging from 4.41 ± 0.06 to 8.08 ± 0.05 mg RUE/g) at *p* ≤ 0.05.

A strong relation between flavonoid content and TPC was observed through statistical analysis. A strong correlation was determined in both natural bee pollen samples (0.813) and samples after hydrolysis with lipase, cellulase, protease, amyloglucosidase, *Viscozyme^®^ L*, and *Clara-diastase* (0.779, 1.000, 0.820, 1.000, 1.000, 1.000, respectively) (see Section 3.5). Such results could be explained by the fact that flavonoids, especially flavones, and flavonols, constitute the largest group of phenolic compounds. Additionally, these biologically active compounds, owing to their chemical structure, are responsible for the antioxidant activity of bee pollen [28,29].

Enzymatic treatments of bee pollen led to a significant (*p* ≤ 0.05) increase in RSA, ranging from 2.0 to 246.4% (Table 4), with the highest activity values obtained after enzymatic hydrolysis with cellulase, *Viscozyme^®^ L* and *Clara-diastase* in comparison to hydrolysis with lipase, protease, and amyloglucosidase. The correlation relationships between total phenolic compounds and RSA, as well as between total flavonoid compounds and RSA, were as follows: correlation coefficients were 0.837 and 0.965 in natural bee pollen, respectively, and within the range of 0.787 to 1.000, and 0.881 to 1.000, respectively, depending on the enzyme used during hydrolysis (see Section 3.5). The very high correlation characteristics confirmed that enzymatic treatment of bee pollen effectively degrades the matrix of the pollen cell wall, releases specific phenolic and flavonoid molecules, and enhances the bioavailability of natural antioxidant sources.

The topic of enzymatic hydrolysis of bee pollen in scientific literature is relatively new, with only a few initial studies having been published. An increase in TPC after enzymatic hydrolysis was also determined by Zuluaga-Dominguez et al. [12], where the amount of phenolics significantly increased by 83 to 106% after hydrolysis with different protease mixtures, flavonoids increased by 85 to 96% and antioxidant activity increased up to 68%. In another study by Zuluaga et al. [4], it was reported that after thermal treatment and enzymatic hydrolysis with *Protamex*™ (endoprotease), bioactive compounds and antioxidant capacity significantly increased by 13 to 14%. During our research, enzymatic hydrolysis with protease led to changes in the amount of TPC by 23 to 94%, TFC by 32 to 57%, and antioxidant capacity by 57 to 134%. The results of other authors align with our study and confirm positive trends of bee pollen hydrolysis with proteases. The main factors influencing the results are the geographical and botanical origins of bee pollen and its freshness, as well as the specificity of the enzyme used for treatment. However, some studies have shown a negative impact of enzymatic hydrolysis on bee pollen compounds. Benavides-Guevara et al. [3] determined a decrease in phenolic compounds (from 15.19 ± 0.58 to 7.00 ± 0.06 mg gallic acid/g) and antioxidant activity (from 0.029 ± 0.001 to 0.006 ± 0.000 mmol Trolox/g in ferric reducing antioxidant power assay; from 0.079 ± 0.014 to 0.015 ± 0.004 mmol Trolox/g in the Trolox equivalent antioxidant capacity assay) after bee pollen enzymatic hydrolysis with *Protamex*^TM^. The discrepancies between these results and our study may be attributed to the use of different technological processes of enzymatic hydrolysis.

### 3.4. Antibacterial Activity in Natural and Enzymatically Hydrolyzed Bee Pollen

The antibacterial activity (AA) of nine bee pollen samples from Sweden, Spain, Netherlands, Italy, Poland, Denmark, Slovakia, Malta, and Lithuania after hydrolysis using lipase, cellulase, protease, amyloglucosidase, *Viscozyme^®^ L* and *Clara-diastase* enzymes was evaluated and the results are presented in Table 5. The inhibition zone of antibacterial activity was compared with the antibacterial activity of ceftazidime (µg CEF/mL).

To our knowledge, this is the first study to evaluate the AA of enzymatically hydrolyzed bee pollen. However, the AA of natural (non-treated) bee pollen has already been analyzed. The AA of natural bee pollen extracts was demonstrated against *Bacillus thuringiensis*, *S. aureus*, *Escherichia coli*, and *Salmonella enterica* by Sawicki et al. [30]. Velásquez et al. [31] determined the antibacterial effect of sixteen bee pollen samples from Chile against *S. aureus*, *E. coli*, *Streptococcus pyogenes*, and *Pseudomonas aeruginosa*. The sensitivity of *E. coli*, *Staphylococcus epidermidis*, and *Candida albicans* to Brazilian bee pollen extracts was also described by Soares de Arruda et al. [32].

A positive effect of enzymatic hydrolysis on bee pollen AA against Gram-positive *S. aureus*, *L. monocytogenes*, and Gram-negative *S. enteritidis*, *S. typhimurium* bacteria cultures was observed. The highest changes in antibacterial activity were determined after enzymatic hydrolysis with cellulase (1.2 to 3.3 times) and *Viscozyme^®^ L* (1.2 to 3.1 times), while the lowest changes were observed with amyloglucosidase (1.1 to 1.6 times). In a previous study [9], lactic acid fermentation was applied, also revealing a positive effect of the treatment on the enhancement of bee pollen antibacterial activity against *Micrococcus luteus*, *S. aureus*, and *E. coli*.

AA was strongly influenced by the strain of pathogenic bacteria. Gram-positive bacteria cultures were found to be 1.1 to 2.5 times more sensitive to natural and enzymatically hydrolyzed bee pollen extracts compared to Gram-negative bacteria. This difference in susceptibility between Gram-positive and Gram-negative bacteria may be attributed to the distinct composition of their cell walls. The membrane of Gram-positive bacteria is rich in low-abundant lipids, which could serve as a specific target for antibacterial compounds found in bee pollen extracts [33]. Similarly, other studies have also analyzed and successfully demonstrated the more effective AA of bee pollen extracts against Gram-positive bacteria cultures. Velasueze et al. [31] and Bakour et al. [34] evaluated the antibacterial impact of bee pollen on *S. aureus*, *E. coli*, and *P. aeruginosa* and both studies reported higher activity of the extracts against Gram-positive bacteria strains.

The antibacterial activity, both before and after enzymatic hydrolysis, was found to exhibit a strong correlation with TPC, TFC, and RSA. The observed trend, characterized by high correlation coefficient values for tested characteristics (see Section 3.5), suggested that the composition and quantity of biologically active compounds have a significant impact on the antibacterial activity of bee pollen. This relationship between the high levels of bioactive compounds, antioxidant content, and AA has also been analyzed and confirmed by other studies [30,31,32,33,34,35].

### 3.5. Chemometric Analysis of the Samples

TPC, TFC, RSA, and AA of natural and enzymatically hydrolyzed bee pollen exhibited a high correlation (see Table 6).

**Figure 6:**
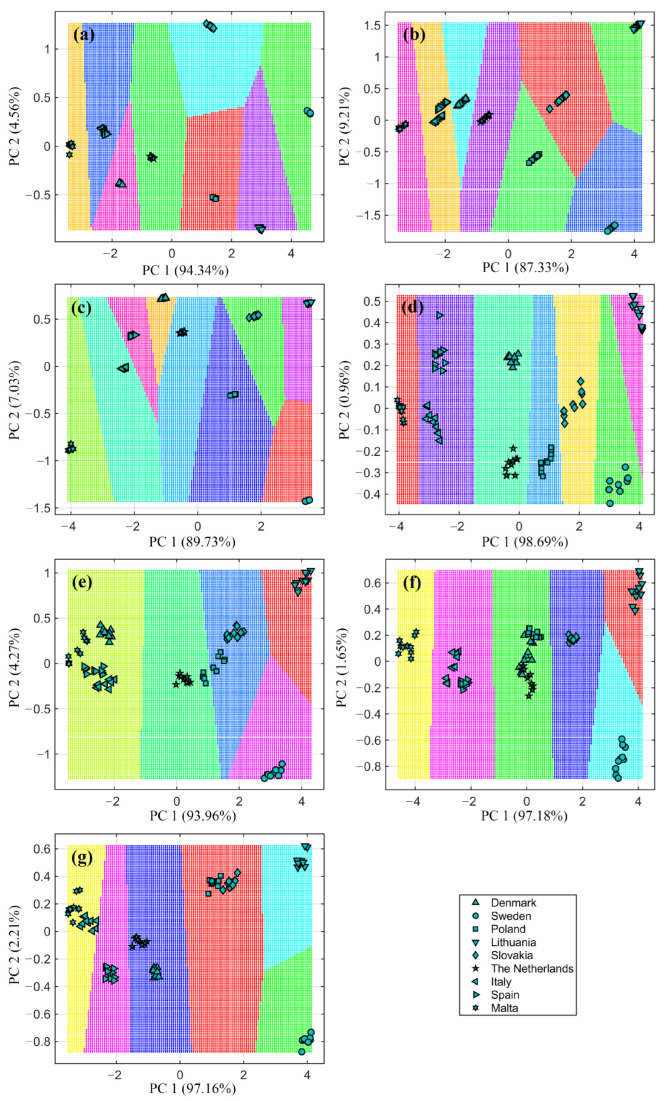
K-means clustering analysis scatter plots presented in the space of principal components of tested bee pollen samples before and after enzymatic hydrolysis. K-means clustering obtained using different measured parameters: (**a**) based on TPC; (**b**) based on TFC; (**c**) based on RSA; (**d**) based on AA against *S. aureus*; (**e**) based on AA against *L. monocytogenes*; (**f**) based on AA against *S. enteritidis*; (**g**) based on AA against *S. typhimurium*.

Principal component analysis (PCA) and clustering analysis were employed to gain a better understanding of the relationship among the results and to determine the similarity of bee pollen samples both before and after the enzymatic hydrolysis process. Two principal components, accounting for 96.54 to 99.65% of the total initial variance in the input variables were suggested by the PCA method. The k-means clustering algorithm was used to visually depict the distribution of various bee pollen samples before and after enzymatic hydrolysis based on the measured parameters, including TPC, TFC, RSA, and AA against *S. aureus*, *L. monocytogenes*, *S. enteritidis*, *S. typhimurium*. Figure 6 shows the grouping of the samples according to the measured parameter.

The clustering results revealed that eight groups were delineated based on the measured characteristic used as an input, specifically TPC and TFC results (see Figure 6a,b). One cluster was formed by samples from Italy and Spain, which exhibited similarity to a sample from Malta. Additionally, the profiles of bee pollen samples from Sweden and Lithuania were distinct from those of Southern European bee pollen. Notably, all bee-collected samples formed individual groups based on RSA results (Figure 6c). Similarly, in terms of TPC and TFC, pollen from Malta was more closely related to samples from Italy, while samples from Sweden were more similar to those from Lithuania, and samples from the Netherlands showed similarities to those from Slovakia and Poland. It could be inferred that the similarity of the tested samples was influenced by their geographical and botanical origins, ranging from northern to southern Europe.

Bee pollen samples were grouped into 5–7 clusters depending on their measured antibacterial activity (Figure 6d–g). Again, distinct groups were observed for Lithuanian and Sweden samples before and after enzymatic hydrolysis. In contrast, samples from South Europe (Malta, Spain, and Italy) and samples from Central Europe (Poland, Slovakia, Denmark, and The Netherlands) exhibited highly similar profiles.

However, the k-means clustering algorithm results exposed that the enzyme used during the hydrolysis process had a more significant impact on clustering than the geographical origin of the bee-collected samples. The clustering results based on TPC, TFC, and RSA, yielded seven clusters before enzymatic hydrolysis. Furthermore, after hydrolysis with amyloglucosidase six groups were identified, while with *Viscozyme^®^ L* and *Clara-diastase*, seven groups were formed. With protease and cellulase, eight groups were suggested, and with lipase, nine groups were observed. The results of antibacterial activity for bee pollen before and after enzymatic hydrolysis revealed 6–7 groups and demonstrated that samples from Central and Northern Europe had distinct profiles, whereas samples from South Europe revealed highly similar profiles.

## 4. Conclusions

The enzymatic hydrolysis process applied to bee pollen and analysis of the obtained results is described in the paper. The evaluation of the optimal amount of enzyme and duration of enzymatic hydrolysis was conducted for six tested enzymes. The results indicated that the optimal parameters are as follows: 3 h 15 min and 175 µL 0.05 units of enzyme per gram of bee pollen using cellulase, *Viscozyme^®^ L* and *Clara-diastase*, while for protease, lipase, and amyloglucosidase the optimal parameters are 3 h 45 min and 200 µL 0.05 units of enzyme per gram of bee pollen.

Statistically significant changes were observed after applying the enzymatic hydrolysis process to bee pollen. The research demonstrated a positive impact of enzymatic hydrolysis on both the antioxidant and antibacterial activity of bee pollen. An increase in TPC (by 1.1 to 2.5 times), TFC (by 1.1 to 3.0 times), RSA (by 1.1 to 3.5 times), and AA against *S. aureus*, *L. monocytogenes*, *S. enteritidis* and *S. typhimurium* (by 1.1–3.3 times) was observed in all tested bee pollen samples after enzymatic hydrolysis. The extent of these changes was dependent on the geographical and botanical origin of bee pollen and the enzyme used for the hydrolysis.

K-means clustering analysis grouped samples into 5–9 clusters based on the measured characteristics, including TPC, TFC, RSA, and AA against four different bacteria. These groupings suggested that the similarity among the tested samples was influenced by their geographical and botanical origins from the north to the south of Europe. However, the results exposed that the enzyme used for the hydrolysis had a more significant impact on clustering results than the geographical origin of the pollen samples. Despite this, enzymatic hydrolysis appears to be a promising method for enhancing the digestibility and bioavailability of this valuable natural bee product.

## Figures and Tables

**Table 1:** Characterization of bee pollen samples.

Country	Location	GPS Coordinates	Collection Date
Spain	Valencia region	39°28′ N 0°22′ W	May 2018
Slovakia	Trnava region	48°22′ N 17°35′ E	Jun 2018
Poland	Bialystok	53°08′ N 23°08′ E	Jul 2018
Lithuania	Šiauliai region, Kuršėnai	55°59′ N 22°55′ E	Aug 2018
Denmark	Alsgarde region	56°04′ N 12°32′ E	Aug 2018
Sweden	Hagfors region	60°02′ N 13°39′ E	Aug 2018
The Netherlands	South Holland, Gouda	52°0′ N 4°42′ E	Aug 2018
Republic of Malta	Northern region, Mellieha	35°57′ N 14°21′ E	Aug 2018
Italy	Bibbiena region	43°42′ N 11°49′ E	2018

**Table 2:** Description of specific enzymes according to the manufacturer.

Enzyme	Type of Enzyme	Temperature	pH Range
*Viscozyme^®^ L*	A mixture of carbohydrases, including arabanase, cellulase, β-glucanase, hemicellulase, and xylanase	40 °C	3.3–5.5
*Clara-diastase*	A mixture of α-amylase, cellulase, invertase, peptidase, phosphatase, and sulfatase	25 °C	6.0–7.0
Lipase	Triacylglycerols hydrolysis	40 °C	7.0–8.0
Protease	Peptide bonds hydrolysis	60 °C	7.0–8.0
Amyloglucosidase	Bonds of oligosaccharides hydrolysis	55 °C	4.0–6.0
Cellulase	Glycosidic linkages hydrolysis	37 °C	4.0–6.0

**Table 3:** Preparation of the samples for the optimal pH value determination.

**Protease [15]**
1 mL 0.65% casein + 0.1 mL enzyme → 10 min → 1 mL 6.1 N trichloroacetic acid + 0.1 mL enzyme → 30 min → 1 mL sodium carbonate + 1 mL Folin-Ciocalteu → 30 min → 660 nm
**Lipase [16]**
0.05 mL 10% Tween + 0.05 mL calcium chloride + 0.25 mL enzyme + 1.15 mL buffer → +37 °C 1 h → 400 nm
**Cellulase [17]**
0.1 mL cellulose + 0.025 mL enzyme → 5 min → 0.25 mL DNS → +100 °C 10 min → 10 min on ice → 546 nm
**Amyloglucosidase, *Viscozyme^®^ L*, *Clara-diastase* [18]**
1% starch + 1 mL enzyme → +40 °C 10 min → 1 mL DNS → +100 °C 5 min → 10 min on ice → 546 nm

**Table 4:** Changes in total phenolic compounds and flavonoid content, and radical scavenging activity of bee pollen before and after enzymatic hydrolysis.

Sample	Total Phenolic Compounds Content, mg RUE/g						
	Before Hydrolysis	Enzyme					
		Protease	Lipase	Cellulase	*Clara-diastase*	*Viscozyme^®^ L*	Amyloglucosidase
Danish	9.82	12.14 *^i^*	10.49 *^i^*	16.67 *^i^*	13.35 *^i^*	15.67 *^i^*	9.97 *^i^*
Swedish	12.67	22.57 *^i^*	17.02 *^i^*	31.67 *^i^*	24.83 *^i^*	29.77 *^i^*	16.17 *^i^*
Polish	11.24	16.23 *^i^*	14.76 *^i^*	23.60 *^i^*	17.85 *^i^*	22.19 *^i^*	14.02 *^i^*
Lithuanian	11.97	17.63 *^i^*	16.99 *^i^*	27.54 *^i^*	19.39 *^i^*	25.89 *^i^*	16.14 *^i^*
Slovak	10.26	19.95 *^i^*	13.47 *^i^*	23.64 *^i^*	21.95 *^i^*	18.35 *^i^*	12.79 *^i^*
Dutch	9.81	14.23 *^i^*	12.62 *^i^*	18.64 *^i^*	15.65 *^i^*	14.76 *^i^*	11.99 *^i^*
Italian	8.66	12.45 *^i^*	10.33 *^i^*	15.59 *^i^*	13.70 *^i^*	11.39 *^i^*	9.81 *^i^*
Spanish	9.09	12.64 *^i^*	10.18 *^i^*	14.54 *^i^*	13.90 *^i^*	11.96 *^i^*	9.67 *^i^*
Maltese	8.07	10.53 *^i^*	9.26 *^i^*	12.57 *^i^*	11.48 *^i^*	9.79 *^i^*	8.84 *^i^*
Mean	10.18	15.37	12.79	20.49	16.90	17.75	12.16
SD	1.53	4.03	2.97	6.46	4.45	6.90	2.81
Min	8.07	10.53	9.26	12.57	11.48	9.79	8.84
Max	12.67	22.57	17.02	31.67	24.83	29.77	16.17
	**Total flavonoid content, mg RUE/g**						
Danish	4.77	6.31 *^i^*	5.49 *^i^*	8.33 *^i^*	6.67 *^i^*	7.83 *^i^*	4.98 *^i^*
Swedish	5.22	8.21 *^i^*	6.25 *^i^*	15.83 *^i^*	12.41 *^i^*	14.88 *^i^*	8.08 *^i^*
Polish	4.87	7.18 *^i^*	6.06 *^i^*	11.80 *^i^*	8.93 *^i^*	11.10 *^i^*	7.01 *^i^*
Lithuanian	6.26	9.67 *^i^*	8.85 *^i^*	13.77 *^i^*	9.70 *^i^*	12.94 *^i^*	8.07 *^i^*
Slovak	5.34	8.21 *^i^*	7.18 *^i^*	11.81 *^i^*	10.96 *^i^*	9.16 *^i^*	6.38 *^i^*
Dutch	4.81	6.74 *^i^*	5.62 *^i^*	9.32 *^i^*	7.82 *^i^*	7.38 *^i^*	5.99 *^i^*
Italian	4.33	6.01 *^i^*	5.15 *^i^*	7.79 *^i^*	6.84 *^i^*	5.69 *^i^*	4.90 *^i^*
Spanish	4.57	6.10 *^i^*	5.20 *^i^*	7.27 *^i^*	6.95 *^i^*	5.98 *^i^*	4.83 *^i^*
Maltese	3.70	5.41 *^i^*	4.99 *^i^*	6.46 *^i^*	5.87 *^i^*	4.93 *^i^*	4.41 *^i^*
Mean	4.87	7.09	6.09	10.26	8.46	8.88	6.07
SD	0.71	1.36	1.24	3.21	2.20	3.45	1.41
Min	3.70	5.41	4.99	6.46	5.87	4.93	4.41
Max	6.26	9.67	8.85	15.83	12.41	14.88	8.08
	**Radical scavenging activity, mg RUE/g**						
Danish	6.11	10.11 *^i^*	9.31 *^i^*	10.42 *^i^*	8.34 *^i^*	9.79 *^i^*	6.23 *^i^*
Swedish	7.38	12.21 *^i^*	11.57 *^i^*	19.79 *^i^*	15.52 *^i^*	18.60 *^i^*	10.11 *^i^*
Polish	6.68	11.21 *^i^*	10.25 *^i^*	14.75 *^i^*	11.16 *^i^*	13.87 *^i^*	8.77 *^i^*
Lithuanian	9.23	14.69 *^i^*	13.62 *^i^*	17.21 *^i^*	12.12 *^i^*	16.18 *^i^*	10.09 *^i^*
Slovak	8.05	13.01 *^i^*	11.79 *^i^*	14.76 *^i^*	13.71 *^i^*	11.46 *^i^*	8.33 *^i^*
Dutch	6.53	10.27 *^i^*	8.86 *^i^*	11.65 *^i^*	9.78 *^i^*	9.22 *^i^*	7.49 *^i^*
Italian	4.55	8.90 *^i^*	6.16 *^i^*	9.74 *^i^*	8.56 *^i^*	7.12 *^i^*	6.13 *^i^*
Spanish	4.73	9.43 *^i^*	7.55 *^i^*	9.09 *^i^*	8.68 *^i^*	7.47 *^i^*	6.04 *^i^*
Maltese	2.33	5.46 *^i^*	5.00 *^i^*	8.07 *^i^*	7.34 *^i^*	6.16 *^i^*	5.50 *^i^*
Mean	6.18	10.59	9.35	12.83	10.58	11.10	7.59
SD	2.07	2.66	2.80	4.01	2.75	4.31	1.76
Min	2.33	5.46	5.00	8.07	7.34	6.16	5.50
Max	9.23	14.69	13.62	19.79	15.52	18.60	10.11

*i*—Observed significant changes after enzymatic hydrolysis are labeled as “*i*” (increased) when *p* ≤ 0.05.

**Table 5:** Changes in antibacterial activity of bee pollen before and after enzymatic hydrolysis.

Sample	Enzyme	Antibacterial Activity, µg CEF/mL			
		Gram-Positive		Gram-Negative	
		*S. aureus*	*L. monocytogenes*	*S. enteritidis*	*S. typhimurium*
Danish	Before hydrolysis	14.26 ± 0.38	13.58 ± 0.31	10.02 ± 0.71	7.19 ± 0.17
	Protease	17.15 ± 0.61 *^i^*	14.26 ± 0.19 *^i^*	14.99 ± 0.46 *^i^*	12.72 ± 0.23 *^i^*
	Lipase	16.25 ± 0.46 *^i^*	14.14 ± 0.78 *^i^*	13.89 ± 0.26 *^i^*	10.03 ± 0.14 *^i^*
	Cellulase	20.15 ± 0.19 *^i^*	19.66 ± 0.26 *^i^*	17.89 ± 0.16 *^i^*	17.72 ± 0.12 *^i^*
	*Clara-diastase*	18.46 ± 0.25 *^i^*	15.40 ± 0.34 *^i^*	15.22 ± 0.60 *^i^*	14.22 ± 0.26 *^i^*
	*Viscozyme^®^ L*	19.15 ± 0.72 *^i^*	18.68 ± 0.46 *^i^*	16.21 ± 0.19 *^i^*	15.22 ± 0.27 *^i^*
	Amyloglucosidase	15.40 ± 0.26 *^i^*	14.01 ± 0.19 *^i^*	11.59 ± 0.61 *^i^*	9.40 ± 0.13 *^i^*
Swedish	Before hydrolysis	20.74 ± 0.80	17.96 ± 0.31	13.26 ± 0.72	13.83 ± 0.30
	Protease	28.54 ± 0.88 *^i^*	29.05 ± 0.34 *^i^*	22.14 ± 0.21 *^i^*	23.26 ± 0.31 *^i^*
	Lipase	26.47 ± 0.89 *^i^*	28.98 ± 0.47 *^i^*	21.87 ± 0.19 *^i^*	22.59 ± 0.33 *^i^*
	Cellulase	31.54 ± 0.72 *^i^*	32.16 ± 0.35 *^i^*	26.14 ± 0.16 *^i^*	28.26 ± 0.19 *^i^*
	*Clara-diastase*	29.47 ± 0.90 *^i^*	30.98 ± 0.67 *^i^*	23.85 ± 0.29 *^i^*	24.16 ± 0.30 *^i^*
	*Viscozyme^®^ L*	29.92 ± 0.47 *^i^*	31.96 ± 0.36 *^i^*	24.49 ± 0.34 *^i^*	27.22 ± 0.14 *^i^*
	Amyloglucosidase	23.16 ± 0.90 *^i^*	19.66 ± 0.82 *^i^*	16.89 ± 0.16 *^i^*	21.72 ± 0.61 *^i^*
Polish	Before hydrolysis	14.46 ± 0.87	18.02 ± 0.83	10.67 ± 0.23	13.76 ± 0.26
	Protease	21.78 ± 0.26 *^i^*	24.17 ± 0.59 *^i^*	14.89 ± 0.16 *^i^*	16.32 ± 0.14 *^i^*
	Lipase	20.19 ± 0.11 *^i^*	22.63 ± 0.82 *^i^*	14.24 ± 0.06 *^i^*	15.14 ± 0.47 *^i^*
	Cellulase	24.15 ± 0.29 *^i^*	26.86 ± 0.75 *^i^*	18.21 ± 0.47 *^i^*	19.52 ± 0.19 *^i^*
	*Clara-diastase*	22.22 ± 0.90 *^i^*	24.66 ± 0.19 *^i^*	15.22 ± 0.38 *^i^*	17.22 ± 0.53 *^i^*
	*Viscozyme^®^ L*	23.15 ± 0.67 *^i^*	25.13 ± 0.46 *^i^*	16.89 ± 0.84 *^i^*	18.67 ± 0.14 *^i^*
	Amyloglucosidase	17.12 ± 0.11 *^i^*	19.66 ± 0.85 *^i^*	13.59 ± 0.67 *^i^*	14.72 ± 0.61 *^i^*
Lithuanian	Before hydrolysis	26.29 ± 0.25	26.95 ± 0.82	19.44 ± 0.68	20.20 ± 0.51
	Protease	29.54 ± 0.90 *^i^*	29.07 ± 0.14 *^i^*	22.24 ± 0.16 *^i^*	22.27 ± 0.14 *^i^*
	Lipase	28.47 ± 0.47 *^i^*	28.16 ± 0.22 *^i^*	21.84 ± 0.29 *^i^*	21.26 ± 0.22 *^i^*
	Cellulase	30.54 ± 0.72 *^i^*	31.16 ± 0.36 *^i^*	25.14 ± 0.16 *^i^*	26.26 ± 0.61 *^i^*
	*Clara-diastase*	29.99 ± 0.18 *^i^*	29.98 ± 0.74 *^i^*	23.46 ± 0.29 *^i^*	23.60 ± 0.21 *^i^*
	*Viscozyme^®^ L*	30.11 ± 0.22 *^i^*	30.96 ± 0.36 *^i^*	24.49 ± 0.34 *^i^*	24.12 ± 0.13 *^i^*
	Amyloglucosidase	27.15 ± 0.13 *^i^*	27.91 ± 0.77 *^i^*	20.19 ± 0.18 *^i^*	20.72 ± 0.41 *^i^*
Slovak	Before hydrolysis	18.84 ± 0.89	22.36 ± 0.29	13.91 ± 0.21	14.94 ± 0.32
	Protease	23.54 ± 0.90 *^i^*	25.06 ± 0.35 *^i^*	18.14 ± 0.16 *^i^*	18.26 ± 0.46 *^i^*
	Lipase	21.47 ± 0.92 *^i^*	23.98 ± 0.46 *^i^*	15.86 ± 0.19 *^i^*	16.61 ± 0.30 *^i^*
	Cellulase	26.54 ± 0.72 *^i^*	27.16 ± 0.38 *^i^*	22.14 ± 0.16 *^i^*	21.26 ± 0.26 *^i^*
	*Clara-diastase*	24.47 ± 0.92 *^i^*	25.98 ± 0.75 *^i^*	18.85 ± 0.29 *^i^*	18.60 ± 0.30 *^i^*
	*Viscozyme^®^ L*	25.12 ± 0.38 *^i^*	26.96 ± 0.38 *^i^*	19.49 ± 0.31 *^i^*	19.22 ± 0.13 *^i^*
	Amyloglucosidase	20.15 ± 0.91 *^i^*	23.54 ± 0.66 *^i^*	14.89 ± 0.15 *^i^*	15.23 ± 0.58 *^i^*
Dutch	Before hydrolysis	12.27 ± 0.72	13.99 ± 0.34	9.04 ± 0.06	6.91 ± 0.34
	Protease	16.91 ± 0.30 *^i^*	21.58 ± 0.14 *^i^*	14.24 ± 0.14 *^i^*	11.62 ± 0.19 *^i^*
	Lipase	15.78 ± 0.47 *^i^*	20.16 ± 0.46 *^i^*	13.63 ± 0.17 *^i^*	9.96 ± 0.35 *^i^*
	Cellulase	22.65 ± 0.11 *^i^*	24.68 ± 0.75 *^i^*	18.89 ± 0.90 *^i^*	14.72 ± 0.61 *^i^*
	*Clara-diastase*	17.99 ± 0.61 *^i^*	22.56 ± 0.61 *^i^*	15.17 ± 0.22 *^i^*	12.72 ± 0.30 *^i^*
	*Viscozyme^®^ L*	20.12 ± 0.92 *^i^*	23.78 ± 0.14 *^i^*	16.89 ± 0.67 *^i^*	13.74 ± 0.46 *^i^*
	Amyloglucosidase	14.15 ± 0.11 *^i^*	19.96 ± 0.26 *^i^*	12.61 ± 0.53 *^i^*	8.81 ± 0.61 *^i^*
Italian	Before hydrolysis	6.57 ± 0.47	11.55 ± 0.30	4.82 ± 0.83	4.42 ± 0.30
	Protease	9.89 ± 0.67 *^i^*	15.34 ± 0.46 *^i^*	8.99 ± 0.05 *^i^*	7.47 ± 0.14 *^i^*
	Lipase	8.44 ± 0.22 *^i^*	13.45 ± 0.34 *^i^*	7.11 ± 0.83 *^i^*	6.37 ± 0.61 *^i^*
	Cellulase	14.89 ± 0.75 *^i^*	20.34 ± 0.14 *^i^*	12.99 ± 0.05 *^i^*	11.47 ± 0.26 *^i^*
	*Clara-diastase*	10.02 ± 0.28 *^i^*	16.45 ± 0.83 *^i^*	9.36 ± 0.54 *^i^*	8.16 ± 0.53 *^i^*
	*Viscozyme^®^ L*	12.15 ± 0.91 *^i^*	19.67 ± 0.74 *^i^*	11.89 ± 0.22 *^i^*	9.72 ± 0.74 *^i^*
	Amyloglucosidase	7.02 ± 0.28 *^i^*	12.45 ± 0.81 *^i^*	6.36 ± 0.54 *^i^*	5.53 ± 0.61 *^i^*
Spanish	Before hydrolysis	7.88 ± 0.72	11.28 ± 0.30	5.79 ± 0.05	4.15 ± 0.30
	Protease	10.77 ± 0.05 *^i^*	14.12 ± 0.13 *^i^*	8.26 ± 0.13 *^i^*	9.80 ± 0.06 *^i^*
	Lipase	9.22 ± 0.18 *^i^*	13.34 ± 0.80 *^i^*	7.61 ± 0.13 *^i^*	8.30 ± 0.26 *^i^*
	Cellulase	13.20 ± 0.61 *^i^*	19.66 ± 0.85 *^i^*	14.89 ± 0.19 *^i^*	13.72 ± 0.74 *^i^*
	*Clara-diastase*	11.15 ± 0.91 *^i^*	15.41 ± 0.16 *^i^*	12.71 ± 0.35 *^i^*	10.44 ± 0.35 *^i^*
	*Viscozyme^®^ L*	12.15 ± 0.41 *^i^*	17.78 ± 0.90 *^i^*	11.65 ± 0.22 *^i^*	12.12 ± 0.26 *^i^*
	Amyloglucosidase	8.46 ± 0.11 *^i^*	12.34 ± 0.81 *^i^*	6.61 ± 0.59 *^i^*	5.31 ± 0.19 *^i^*
Maltese	Before hydrolysis	3.94 ± 0.11	11.02 ± 0.89	2.87 ± 0.59	3.89 ± 0.51
	Protease	6.27 ± 0.46 *^i^*	13.99 ± 0.46 *^i^*	4.04 ± 0.90 *^i^*	6.26 ± 0.14 *^i^*
	Lipase	5.24 ± 0.19 *^i^*	12.22 ± 0.61 *^i^*	3.22 ± 0.67 *^i^*	5.98 ± 0.74 *^i^*
	Cellulase	12.27 ± 0.46 *^i^*	16.99 ± 0.46 *^i^*	9.04 ± 0.54 *^i^*	9.26 ± 0.06 *^i^*
	*Clara-diastase*	7.25 ± 0.12 *^i^*	14.48 ± 0.84 *^i^*	7.09 ± 0.67 *^i^*	7.10 ± 0.13 *^i^*
	*Viscozyme^®^ L*	8.50 ± 0.18 *^i^*	15.66 ± 0.16 *^i^*	8.89 ± 0.12 *^i^*	8.72 ± 0.23 *^i^*
	Amyloglucosidase	4.64 ± 0.26 *^i^*	11.82 ± 0.80 *^i^*	3.09 ± 0.67 *^i^*	4.70 ± 0.73 *^i^*

*i*—Observed significant changes after enzymatic hydrolysis are labeled as “*i*” (increased) when *p* ≤ 0.05.

**Table 6:** Correlation coefficients between total phenolic compounds content (TPC), total flavonoid content (TFC), radical scavenging activity (RSA), and antibacterial activity (AA).

Criteria	Natural				With Protease				With Lipase				With Cellulase			
	TPC	TFC	RSA	AA	TPC	TFC	RSA	AA	TPC	TFC	RSA	AA	TPC	TFC	RSA	AA
TPC	1	0.813	0.837	0.842	1	0.820	0.787	0.890	1	0.779	0.886	0.954	1	1.000	1.000	0.957
TFC		1	0.965	0.940		1	0.939	0.926		1	0.881	0.801		1	1.000	0.956
RSA			1	0.926			1	0.898			1	0.939			1	0.956
AA				1				1				1				1
**Criteria**	**With *Clara-diastase***				**With *Viscozyme^®^ L***				**With amyloglucosidase**							
	**TPC**	**TFC**	**RSA**	**AA**	**TPC**	**TFC**	**RSA**	**AA**	**TPC**	**TFC**	**RSA**	**AA**				
TPC	1	1.000	1.000	0.893	1	1.000	1.000	0.947	1	1.000	0.998	0.916				
TFC		1	1.000	0.892		1	1.000	0.946		1	0.998	0.916				
RSA			1	0.891			1	0.946			1	0.926				
AA				1				1				1				

## Data Availability

Not applicable.

## References

[1] Denisow B., Denisow-Pietrzyk M. (2016). Biological and Therapeutic Properties of Bee Pollen: A Review. J. Sci. Food Agric..

[2] Mărgăoan R., Strant M., Varadi A., Topal E., Yücel B., Cornea-Cipcigan M., Campos M.G., Vodnar D.C. (2019). Bee Collected Pollen and Bee Bread: Bioactive Constituents and Health Benefits. Antioxidants.

[3] Benavides-Guevara R.M., Quicazan M.C., Ramirez-Toro C. (2017). Digestibility and Availability of Nutrients in Bee Pollen Applying Different Pretreatments. Ing. Compet..

[4] Zuluaga C.M., Serrato-Bermudez J.C., Quicazan M.C. (2015). Bee-pollen Structure Modification by Physical and Biotechnological Processing: Influence on the Availability of Nutrients and Bioactive Compounds. Chem. Eng. Trans..

[5] Rzepecka-Stojko A., Stojko J., Kurek-Górecka A., Górecki M., Kabała-Dzik A., Kubina R., Moździerz A., Buszman E. (2015). Polyphenols from Bee Pollen: Structure, Absorption, Metabolism and Biological Activity. Molecules.

[6] Dong J., Gao K., Wang K., Xu X., Zhang H. (2015). Cell Wall Disruption of Rape Bee Pollen Treated with Combination of Protamex Hydrolysis and Ultrasonication. Food Res. Int..

[7] Wu W., Qiao J., Xiao X., Kong L., Dong J., Zhang H. (2021). In vitro and In vivo Digestion Comparison of Bee Pollen with or Without Wall-disruption. J. Sci. Food Agric..

[8] Xiang X., Sun S., Dong J., Zhang H. (2009). Breaking the Cells of Rape Bee Pollen and Consecutive Extraction of Functional Oil with Supercritical Carbon Dioxide. Innov. Food Sci. Emerg. Technol..

[9] Kaškonienė V., Adaškevičiūtė V., Kaškonas P., Mickienė R., Maruška A. (2020). Antimicrobial and Antioxidant Activities of Natural and Fermented Bee Pollen. Food Biosci..

[10] Filannino P., Di Cagno R., Gambacorta G., Tlais A.Z.A., Cantatore V., Gobbetti M. (2021). Volatilome and Bioaccessible Phenolics Profiles in Lab-scale Fermented Bee Pollen. Foods.

[11] Uțoiu E., Matei F., Toma A., Diguță C.F., Ștefan L.M., Mănoiu S., Vrăjmașu V.V., Moraru I., Oancea A., Israel-Roming F. (2018). Bee Collected Pollen with Enhanced Health Benefits, Produced by Fermentation with a Kombucha Consortium. Nutrients.

[12] Zuluaga-Domínguez C., Castro-Mercado L., Cecilia Quicazán M. (2019). Effect of Enzymatic Hydrolysis on Structural Characteristics and Bioactive Composition of Bee-pollen. J. Food Process. Preserv..

[13] Adaškevičiūtė V., Kaškonienė V., Barčauskaitė K., Kaškonas P., Maruška A. (2022). The Impact of Bacterial Fermentation on Bee Pollen Polyphenolic Compounds Composition and Antioxidant Activity. Antioxidants.

[14] Adaškevičiūtė V., Kaškonienė V., Kaškonas P., Barčauskaitė K., Maruška A. (2019). Comparison of Physicochemical Properties of Bee Pollen with Other Bee Products. Biomolecules.

[15] Merck Universal Protease Activity Assay: Casein as a Substrate. https://www.sigmaaldrich.com/technical-documents/protocols/biology/proteaseactivity-assay.html.

[16] Margesin R., Feller G., Hämmerle M., Schinner F. (2002). A Colorimetric Method for the Determination of Lipase Activity in Soil. Biotechnol. Lett..

[17] Afzal M., Qureshi M.Z., Ahmed S., Khan M.I., Ikram H., Asma A., Qureshi N.A. (2019). Production, Purification and Optimization of Cellulase by *Bacillus licheniformis* HI-08 Isolated from the Hindgut of Wood-feeding Termite. Int. J. Agric. Biol..

[18] Mukhtar H., Ikram-Ul-Haq (2012). Concomitant Production of Two Proteases and Alpha-amylase by a Novel Strain of *Bacillus subtilis* in a Microprocessor Controlled Bioreactor. Braz. J. Microbiol..

[19] Alwazeer D., Sally D.H.A.M. (2019). Presumptive Relationship between Oxidoreduction Potential and Both Antibacterial and Antioxidant Activities of Herbs and Spices: Oxidoreduction Potential as a Companion Tool for Measuring the Antioxidant Activity. Not. Bot. Horti. Agrobot. Cluj-Napoca.

[20] Adaškevičiūtė V., Kaškonienė V., Maruška A. Optimization of Conditions for Bee Pollen Lactic Acid Fermentation. Proceedings of the CYSENI 2021: The 17th International Conference of Young Scientists on Energy Issues.

[21] Kaškonienė V., Kaškonas P., Maruška A. (2015). Volatile Compounds Composition and Antioxidant Activity of Bee Pollen Collected in Lithuania. Chem. Pap..

[22] Uchida V.H., dos Santos E.S. (2020). Production of Cellulases by *Aspergillus niger* IOC 3998 by Means of Solid-State Fermentation (SSF) using as Substrate the Chestnut Seed (*Terminalia catappa Linn.*). J. Waste Resour. Reprocess..

[23] Malik S., Iftikhar T., Haq O., Manzoor K.I. (2013). Process Optimization for Amyloglucosidase by a Mutant Strain of *Aspergillus niger* in Stirred Fermenter. Pak. J. Bot..

[24] Ma X., Kexin Z., Yonggang W., Ebadi A.G., Toughani M. (2021). Optimization of Low-Temperature Lipase Production Conditions and Study on Enzymatic Properties of *Aspergillus niger*. Iran. J. Chem. Chem. Eng..

[25] Otroshi B., Anvari M., Shariarinour M. (2014). Study on Activity and Stability of Proteases from *Bacillus sp*. Produced by Submerged Fermentation. Int. J. Adv. Biol. Biom. Res..

[26] Araújo J.S., Chambó E.D., Costa M.A.P.D.C., Da Silva S.M.P.C., De Carvalho C.A.L., Estevinho L.M. (2017). Chemical Composition and Biological Activities of Mono- and Heterofloral Bee Pollen of Different Geographical Origins. Int. J. Mol. Sci..

[27] He X., Dai J., Wu Q. (2016). Identification of Sporopollenin as the Outer Layer of Cell Wall in Microalga *Chlorella protothecoides*. Front. Microbiol..

[28] Mutha R.E., Tatiya A.U., Surana S.J. (2021). Flavonoids as Natural Phenolic Compounds and Their Role in Therapeutics: An Overview. Future J. Pharm. Sci..

[29] Ghasemzadeh A., Ghasemzadeh N. (2011). Flavonoids and Phenolic Acids: Role and Biochemical Activity in Plants and Human. J. Med. Plant Res..

[30] Sawicki T., Starowicz M., Kłębukowska L., Hanus P. (2022). The Profile of Polyphenolic Compounds, Contents of Total Phenolics and Flavonoids, and Antioxidant and Antimicrobial Properties of Bee Products. Molecules.

[31] Velásquez P., Rodríguez K., Retamal M., Giordano A., Valenzuela L., Montenegro G. (2017). Relation Between Composition, Antioxidant and Antibacterial Activities and Botanical Origin of Multi-floral Bee Pollen. J. Appl. Bot. Food Qual..

[32] Soares de Arruda V.A., dos Santos A.V., Sampaio D.F., Araújo E.S., de Castro Peixoto A.L., Estevinho M.L., de Almeida-Muradian L.B. (2021). Brazilian Bee Pollen: Phenolic Content, Antioxidant Properties and Antimicrobial Activity. J. Apic. Res..

[33] Blair J.M., Webber M.A., Baylay A.J., Ogbolu D.O., Piddock L.J. (2015). Molecular Mechanisms of Antibiotic Resistance. Nat. Rev. Microbiol..

[34] Bakour M., Laaroussi H., Ousaaid D., Oumokhtar B., Lyoussi B. (2021). Antioxidant and Antibacterial Effects of Pollen Extracts on Human Multidrug-Resistant Pathogenic Bacteria. J. Food Qual..

[35] Didaras N.A., Karatasou K., Dimitriou T.G., Amoutzias G.D., Mossialos D. (2020). Antimicrobial Activity of Bee-Collected Pollen and Beebread: State of the Art and Future Perspectives. Antibiotics.

